# Lignin Distribution on Cell Wall Micro-Morphological Regions of Fibre in Developmental *Phyllostachys pubescens* Culms

**DOI:** 10.3390/polym14020312

**Published:** 2022-01-13

**Authors:** Bo Liu, Lina Tang, Qian Chen, Liming Zhu, Xianwu Zou, Botao Li, Qin Zhou, Yuejin Fu, Yun Lu

**Affiliations:** Research Institute of Wood Industry, Chinese Academy of Forestry, Beijing 100091, China; liubo@criwi.org.cn (B.L.); 18211090798@163.com (L.T.); chenqian0610@126.com (Q.C.); izhulm@caf.ac.cn (L.Z.); xwzou@caf.ac.cn (X.Z.); botaoLi@163.com (B.L.); zhouqin567@sina.com (Q.Z.)

**Keywords:** *Phyllostachys pubescens*, fibre, lignification, micro-morphological regions of cell wall, syringyl lignin, guaiacyl lignin, visible-light spectrophotometry

## Abstract

Bamboo is a natural fibre reinforced composite with excellent performance which is, to a certain extent, an alternative to the shortage of wood resources. The heterogeneous distribution and molecular structure of lignin is one of the factors that determines its performance, and it is the key and most difficult component in the basic research into the chemistry of bamboo and in bamboo processing and utilization. In this study, the distribution of lignin components and lignin content in micro-morphological regions were measured in semi-quantitative level by age and radial location by means of visible-light microspectrophotometry (VLMS) coupled with the Wiesner and Maule reaction. There as guaiacyl lignin and syringyl lignin in the cell wall of the fibre. Lignin content of the secondary cell wall and cell corner increased at about 10 days, reached a maximum at 1 year, and then decreased gradually. From 17 days to 4 years, the lignin content of the secondary cell wall in the outer part of bamboo is higher than that in the middle part (which is, in turn, higher than that in the inner part of the bamboo). VLSM results of the micro-morphological regions showed that bamboo lignification developed by aging. Guaiacyl and syringl lignin units can be found in the cell wall of the fibre, parenchyma, and vessel. There was a difference in lignin content among different ages, different radial location, and different micro-morphological regions of the cell wall. The fibre walls were rich in guaiacyl lignin in the early stage of lignification and rich in syringyl units in the later stage of lignification. The guaiacyl and syringyl lignin deposition of bamboo green was earlier than that of the middle part of bamboo culm, and that of the middle part of bamboo culm was earlier than that of bamboo yellow. The single molecule lignin content of the thin layer is higher than that of thick layers, while the primary wall is higher than the secondary cell wall, showing that lignin deposition is consistent with the rules of cell wall formation. The obtained cytological information is helpful to understand the origin of the anisotropic, physical, mechanical, chemical, and machining properties of bamboo.

## 1. Introduction

Bamboo is a profuse, long-lasting resource and the quickest developing and most adaptable plant on Earth. Bamboo has a wide range of applications. Due to its sustainability, extraordinary growth rate, accessibility, light weight, high mechanical strength, and good toupghness, it has been widely used as structural materials and bio-composites for industrial applications [1,2]. Bamboo is also considered to be an important source of biofuels and biochemical production [3]. Many excellent properties of bamboo stem are governed by the properties of the cell wall, which can be described in terms of the sub microstructure of cell wall and the localization of cell wall components of cellulose, hemicellulose, and lignin.

Being the most abundant biopolymer on earth, lignin shows beneficial structural properties [4]. Lignin is an amorphous polymer of benzene linked by the ether bond of propane and carbon-carbon (C-C) bond [5]. Lignin has various unique characteristics, including biocompatibility, antioxidant, antimicrobial, redox activity, etc. [6]. Lignin can be used in various industrial applications, including bio-fuels, chemicals, polymers, etc. Lignin can also be utilized in biomedical applications, such as drug delivery. However, these applications depend on the source, chemical modification, and physico-chemical properties of lignin [7]. An important role of lignin in the wood cell wall is to function as a cross-linking matrix between moisture sensitive cellulose and hemicelluloses. Thereby, lignin is considered to be a special compound closely related to the mechanical strength of cell wall [8,9]. Wood parenchyma cells do not generally contain lignin, but in bamboo culms, lignin widely exists in all kinds of lignified bamboo tissues [10]. It is the important component of the fibre cell wall, parenchyma cell wall, and vessel cell wall [11]. Therefore, understanding the structural distribution of bamboo lignin on the cell wall, especially “seeing” the micro structure of bamboo in the sense of chemical element distribution, is of great significance for making full use of bamboo lignin resources [12].

Studies on lignin in bamboos have been carried out in species of the genus *Phyllostachys* characteristically lacking free fibre strands [13,14], and possessing free fibre strands is associated with the vascular bundle [9,15]. The studies mainly focused on the lignification progress [16,17], various lignin content [10], peroxidase in lignification [18], lignin structure [4,15], and lignin industrial application [5], etc. However, the heterogeneous distribution and molecular structure of lignin are one of the factors that determine the performance of bamboo. Considering the heterogeneous distribution of lignin and the quantitative analysis of the micro morphological region of cell wall from the perspective of bamboo processing and utilization performance is of great significance for the basic research of bamboo chemistry and bamboo processing and utilization.

The vascular bundles of *Phyllostachys pubescens* are typically of the type III, which has a central vascular strand with four small fibre caps, one adjacent to the protoxylem, one to the phloem and one to each of the two large metaxylem vessels [19,20]. The in situ distribution and the content of lignin component within the fiber located at bamboo green, bamboo timber and bamboo yellow, and also located at different cell wall micro-morphological regions within fibre cap on radial and longitudinal location were visualized by complementary microscopy techniques (visible-light microspectrophotometry) coupled with the biochemical method (Wiesner and Maule reactions), especially in different cell wall growth stages. The obtained cytological information is helpful to understand the origin of the anisotropic, physical, mechanical, chemical, and machining properties of bamboo.

## 2. Materials and Methods

### 2.1. Bamboo Samples

12- and 17-day-old immature bamboo shoots and 1- and 4-year-old mature bamboo culms of *Phyllostachys edulis* Carr. Lehaie were harvested on 23 April 2007 in Miaoshanwu Bamboo Garden (29°44′–30°12′ N, 119°25′–120°09′ E and 20 m altitude) of Semitropical Forestry Institute, Chinese Academy of Forestry, Zhejiang Province, China. Blocks of about 2 cm along the grain, including the green and yellow parts of bamboo culm, were cut from the middle part of the 13th internode above the ground and preserved in Formaldehyde-acetic acid-ethanol Fixative (FAA). 3 blocks for each bamboo age were taken as repetition.

### 2.2. Sectioning and Microscopy

Transverse sections of 20 μm in thickness, including the whole radial culm wall, were cut with a sliding microtome (Yamato Kohki TU-213, Saitama, Japan). All sections were observed directly under light microscope (Olympus BX50F4, Tokyo, Japan) for checking integrity of vascular bundles. Three sections from each sample block as repetition were used for detection of autofluorescence of cell walls by fluorescence microscopy before and after chemical treatments. The average value of nine sections from three sample blocks was as the value of lignin content.

### 2.3. Mäule Reaction, Wiesner Reaction and Visible-Light Spectrophotometry

**Figure 1:**
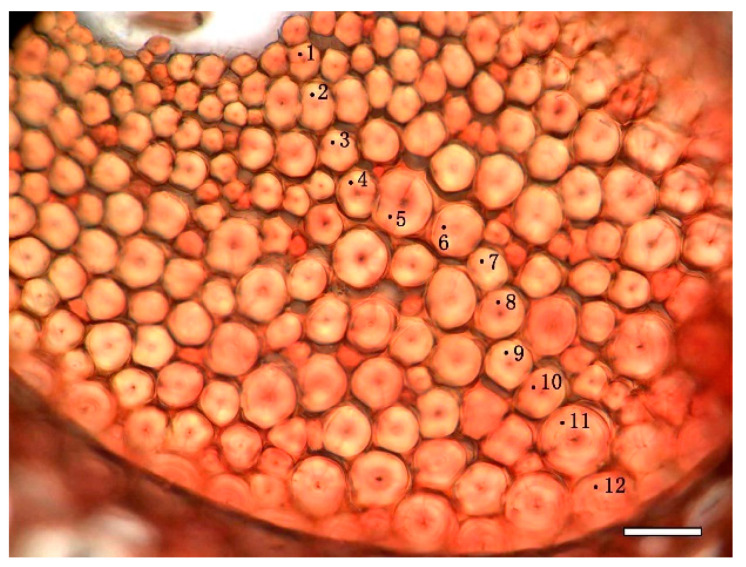
Testing points on different cell wall layers from inside to outside of the outer fibre cluster in the vascular bundle (four-year-old bamboo, the outer part of bamboo, Mäule reaction (Bar = 20 μm)).

**Figure 2:**
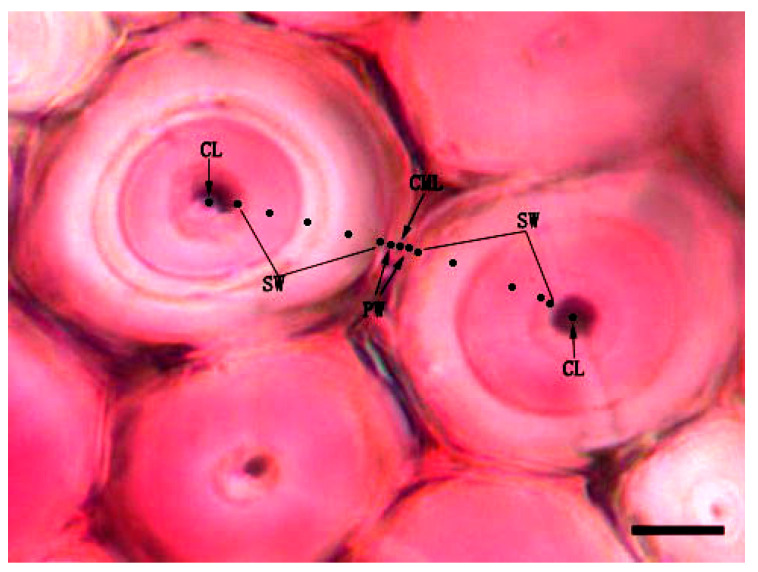
Testing points on different micro-morphological regions of fibre wall in lignin content test—four-year-old, the outer part of bamboo, Wiesner reaction (Bar = 5 μm). CML, cell middle lamella; PW, primary wall; SW, secondary wall; CL, cell lumen.

Mäule and Wiesner reactions were classically applied to stain the syringylpropane and guaiacylpropane units of the cell wall lignin, respectively. After rinsing by distilled water, half of the transverse sections were treated in 1% KMnO_4_ for 5 min followed by three washing in distilled water, then immersed in 3% HCl for 1 min and washed again in distilled water before being mounted in 29% ammonia and immediately observed by fluorescence microscope [21]. Other transverse sections were treated with 2% phloroglucinol in 95% ethanol for 5 min and mounted in 6 M HCl [17]. Absorption spectra from 450 nm to 650 nm at interval of 5 nm were measured with a visible-light microspectrophotometer (UNIVAR, Austria, spot size: 1.0 μm, band width: 10 μm) on the sections after Mäule and Wiesner reactions. Measurements were repeated for three times at every wavelength for the measuring points in each micro-morphological regions of cell wall. Visible-light absorption value of fibre was measured on four different levels: (1) different bamboo ages; (2) different radial positions of bamboo culms, including the green part, the middle part and the yellow part of bamboo culm; (3) different positions of vascular bundle fiber cap, from inner-side to outer-side of fibre cap adjacent to phloem in vascular bundles (Figure 1, four-year-old fibre in Mäule reaction); and (4) different positions of cell micro-morphological regions between two adjacent cells (Figure 2), including cell corner (CC), compound middle lamella (CML), the primary wall (PW), and the layers of secondary wall (SW).

## 3. Results and Discussion

### 3.1. Histochemical Staining of the Cell Walls and Determination of Lignin Components

In histochemical staining, chemicals mainly reacted with syringyl lignin of cell wall in Mäule reaction. Then, the cell walls of dicotyledon wood fibres are stained into amaranth, but the cell walls of gymnosperm wood fibres are stained brown [22]. The Wiesner reaction has universal applicability to guaiacyl and syringyl lignin, which cell walls were stained into red in Wiesner reaction. In this study, fibres of *Phyllostachys pubescens* displayed light brown to dark brown in Mäule reaction, and pink or red in Wiesner reaction.

The layering of fibre generally alternate thick and thin layers with different fibrillar orientation [22]. To find out whether there is the difference of lignin content between thin layer and thick layer of the secondary wall, as an illustration, the secondary wall of four-year-old fibre in Wiesner reaction was divided into two micro-morphological regions for research, which were marked as B (the thin layer of the secondary wall) and H (the thick layer of the secondary wall). The detailed measurements were detected on micro-morphological regions of double cell walls between two fibre lumen. From one side to the other side, visible-light absorption value was measured gradually on B, PW, CML, PW and H in turn (Figure 2, four-year-old fibre in Mäule reaction). Due to the temporary nature of the colour reactions, all of the measurements were performed within 10 min. Then visible-light absorption spectra taken were averaged to give the mean spectrum, from which the mean absorbance at each developmental stage of the secondary wall was obtained.

**Figure 3:**
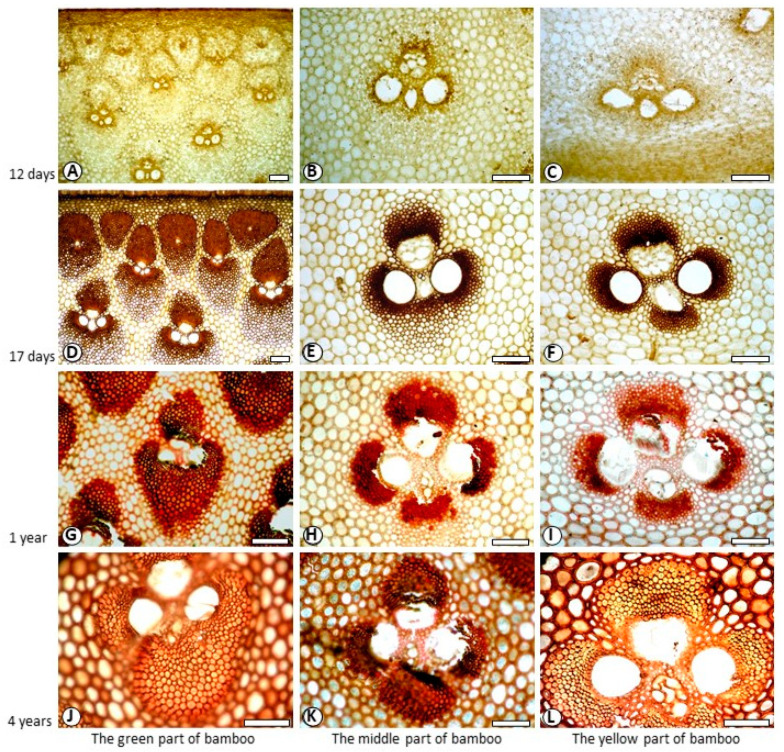
Fibre staining in Mäule reaction. Bar = 100 μm. (**A**) 12-day-old, the green part of bamboo; (**B**) 12-day-old, the middle part of bamboo, (**C**) 12-day-old, the yellow part of bamboo; (**D**) 17-day-old, the green part of bamboo; (**E**). 17-day-old, the middle part of bamboo, (**F**). 17-day-old, the yellow part of bamboo; (**G**). 1-year-old, the green part of bamboo; (**H**). 1-year-old, the middle part of bamboo; (**I**). 1-year-old, the middle part of bamboo; (**J**). 4-year-old, the green part of bamboo; (**K**). 4-year-old, the middle part of bamboo; (**L**). 4-year-old, the yellow part of bamboo.

**Figure 4:**
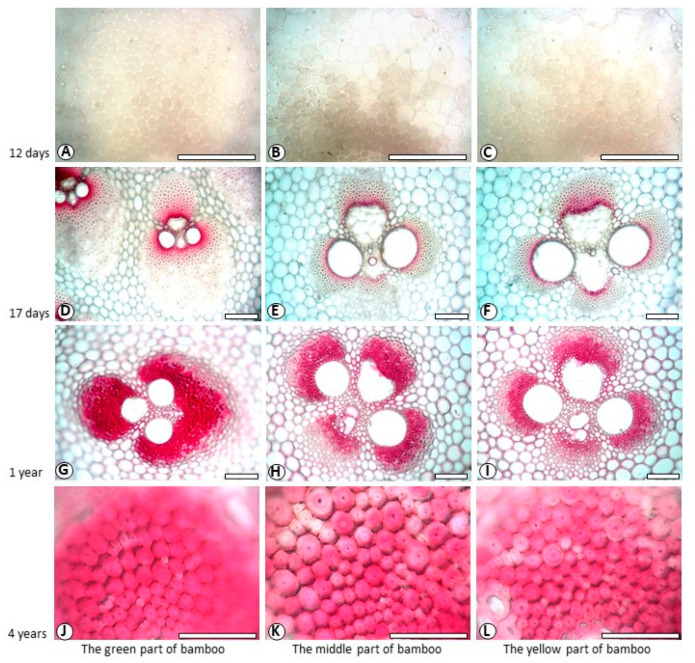
Fibre staining in Weisner reaction. Bar = 100 μm. (**A**) 12-day-old, the green part of bamboo; (**B**) 12-day-old, the middle part of bamboo, (**C**) 12-day-old, the yellow part of bamboo; (**D**) 17-day-old, the green part of bamboo; (**E**). 17-day-old, the middle part of bamboo, (**F**). 17-day-old, the yellow part of bamboo; (**G**). 1-year-old, the green part of bamboo; (**H**). 1-year-old, the middle part of bamboo; (**I**). 1-year-old, the middle part of bamboo; (**J**). 4-year-old, the green part of bamboo; (**K**). 4-year-old, the middle part of bamboo; (**L**). 4-year-old, the yellow part of bamboo.

Figure 3 and Figure 4 showed the color reactions of fibre cell walls in Mäule and Wiesner reactions. Since the fibers in the vascular bundle of bamboo over one-year-old are extremely tough, when sliced with sliding microtome, the quality of slicing is not high, and the fiber cell wall is particularly easy to crack. The high strength and toughness of fibers often causes the edge of slicing blade to crack. The damage rate of slices increased with the increase of bamboo age. Cell wall of 12-day-old fibre did not show characteristic colours in both reactions (Figure 3A–C, Figure 4A–C). Cell wall of 17-day-old fibre showed back-brown in Mäule reaction (Figure 3D–F), especially in the fibres near the centre of the vascular bundle, according to the mature degree of fibre cell wall. However, cell wall of 17-day-old fibre in Weisner reaction showed pink colour on the area near the centre of the vascular bundle, indicating that fibre began lignification 17 days from shooting. Both one-year-old and four-year-old fibre showed brown in Mäule reaction (Figure 3G–I), and reddish pink in Wiesner reaction (Figure 4G–I), wherever in the green or yellow part of bamboo. This reflected the different developmental progress and distribution of guaiacyl and syringyl lignin. The positive reactions with Mäule and Weisner reagents demonstrated that both the guaiacyl (G) and syringyl (S) units were the components of fibre lignin. This accords with the general view which angiosperm plants contained abundant G and S units in lignin. Lybeer and Koch [23] found that the lignin of fibre in *Gigantochloa levis* and *Phyllostachys viridiglaucescens* (Carr.) Riv. & Riv. culms had G, S and p-hydroxyphenypropane (H) units. That was the three diferent phenyl propane monomers: coniferyl alcohol, syringyl alcohol, and coumaryl alcohol precursors. In softwoods, coniferyl alcohol is higher. In hardwoods, syringyl alcohol is abundant, and in crops and grasses, coumaryl alcohol is dominant [5,24,25]. The different proportions of these three monomers determine the important biological characteristics of plants, such as different rigidity and stiffness characteristics, water absorption, antifungal and insecticidal characteristics. Although there exists a little H lignin in monocotyledon plants, it has still no effective histochemical method for its detection and visual microscopic observation.

### 3.2. Visible-Light Absorption Spectra

**Figure 5:**
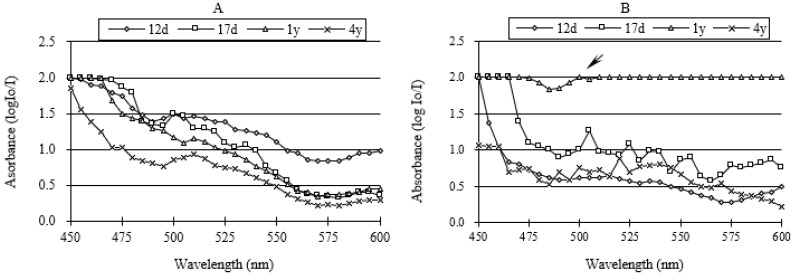
Visible-light absorption spectra for Mäule (**A**) and Wiesner (**B**) reactions of fibre in different ages. 12d, 12-day-old; 17d, 17-day-old; 1y, 1-year-old; 4y, 4-year-old.

Visible light absorption spectra varied remarkably with culm age. Figure 5A,B showed the visible-light spectra taken on the thickest layer of fibre in different ages after Mäule and Weisner reactions. The spectrum exhibited respectively the absorption peak, summarized in Table 1. From 12 days to 4 years, absorption spectrum revealed clear peaks at 500 nm and 510 nm in the Mäule reaction. Absorption maxima of the 12-day-old fibre, 17-day-old fibre, and 4-year-old fibre were at 515 nm, 505 nm, and 520 nm, respectively, in the Weisner reactions. However, the spectra of four-year-old fibre had an unclear shoulder. Most of the absorption values were near the upper limit of the reference range, except a little peak at 500 nm. So, 500 nm was selected (arrowed in Figure 5B) for the detection of four-year-old fibre. Then, the absorption values on different micro-regions of fibre cell wall were measured at corresponding absorption spectra peak value.

### 3.3. Variation of Lignin Content of SW and CC in Different Ages

**Figure 6:**
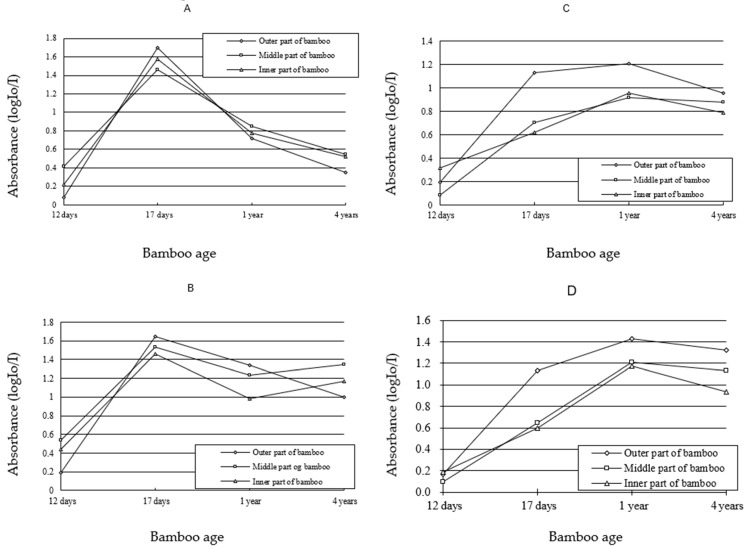
Change curve of absorbance of Mäule and Wiesner reaction in fibre secondary wall and cell corner during lignification process—(**A**) Secondary wall, Mäule reaction; (**B**) secondary wall, Wiesner reaction; (**C**): cell corner, Mäule reaction; (**D**): cell corner, Wiesner reaction.

For SW, the absorbance values were very low in both of Mäule and Wiesner reactions, when fibre was 12-day-old (Figure 6A,B). This was consistent with the results of Figure 3 and Figure 4, and indicated that fibre was in the swelling stage. It was the beginning of fibre lignification. When the fibre was 17-day-old, the curve showed the absorbance value of SW ascended to the top, before descending in the Mäule reaction. Since the Mäule reaction was the characteristic reaction to distinguish syringyl lignin [26], the content of syringyl lignin was the most abundance in SW of 17-day-old fibre and gradually decreased when growth was occurring (Figure 6A). The value was nearly equaled to the level of shooting age when fibre was four years old. In the Wiesner reaction, the content of lignin in SW increased at one period, and reached the absorption maximum at one year before decreasing gradually (Figure 6B). Since both guaiacyl and syringyl lignin react with Wiesner reagents, the absorbance value indicates the both lignin units from the stain colour by means of visible-light microspectrophotometry. It was found that the content of guaiacyl lignin increased from 17-day-old to 1-year-old. Comparing the results of Wiesner and Mäule reaction, it was concluded that guaiacyl lignin was increasing when syringyl lignin was decreasing. The content of both guaiacyl and syringyl lignin decreased a little after one year.

For cell corner, the variation trends of lignin components were generally similar to those of secondary wall in different ages. The difference was that the content of syringyl lignin on cell corner was always higher than that on secondary wall in the middle and yellow parts of bamboo culms in Mäule reaction (Figure 6C) when fibre was four years old. So, the rapid decreasing of guaiacyl lignin must be the reason that both content of lignin units decreased in the middle and yellow parts of bamboo culm from one-year-old to four-year-old samples in contrast with the results of Wiesner reaction.

In the initial period of lignification, the lignin content of fibre cell wall was low. However, it enhanced rapidly following cell development and fibre became one cell type containing the highest concentration of lignin. It owed to different functions of cells during bamboo growth process. As mechanical supporting tissue, fibre cells deposited lignin rapidly in order to improve the mechanics property and the resistance to outside attacks. This is consistent with the literature “the difference of lignin components reflects the difference of cell functions” [10]. In particular, the variation in the modulus was mainly due to the variation in the cell wall lignification level and its composition [12]. The observed difference in the modulus of elasticity between developing and fully lignified cell walls is due to the filling of the spaces with lignin and an increase in the packing density of the cell wall during lignification [27]. In addition, the results of lignin heterogeneity researched in *Quercus mongolica* latewood indicated that guaiacyl lignin was abundant in fibre secondary wall during the initial period of lignification [28]. However, the content and proportion of syringyl lignin would increase gradually with the development of lignification. Syringyl lignin would become the main unit in bamboo culm. Fukushima and Terashima [29] studied the lignin components of sugarcane and rice by means of UV microspectrophotometry. They suggested guaiacyl lignin is the main body in secondary wall of protoxylem vessel. In this study, syringyl lignin was abundant in fibre secondary wall of *Phyllostachys pubescens* during the initial stage of lignification, but decreased in the middle and last stages of lignification, accompanying with the increasing of guaiacyl lignin. These results are different from the research results of *Quercus mongolica*, sugarcane and rice. This may be due to the difference of plant genus.

### 3.4. Variation of Lignin Content of SW and CC in Different Radial Location

The results of Mäule reaction showed that syringyl lignin of SW deposited more in the green and yellow parts of bamboo culm than in the middle part of bamboo culm when fibre was 17 day sold. The sequence of lignin content was the green part, yellow part and middle part of bamboo culm. However, syringyl lignin of SW deposited more in the middle part of bamboo culm than in the other part of bamboo culm, when the fibre was twelve days old, one year old or four year sold. The sequence of lignin content was the middle part, yellow part and green part of bamboo culm. The results of Wiesner reaction indicated that the whole lignin of syringyl and guaiacyl units was the most abundant in yellow part of bamboo culm, and the lesser abundant in green part of bamboo culm, when fibre was 12-day-old. While it varied to the most abundant in the green part of bamboo culm, and the lesser abundant in the middle part of bamboo culm, when fibre was after 12-day-old.

There was some difference of lignin content on CC with SW of fibre. When fibre was 12-day-old and 4-year-old, the sequence of syringyl lignin content on CC was the middle part, yellow part and green part of bamboo culm. But when fibre was 17-day-old and 1-year-old, the sequence of syringyl lignin content on CC was the green part, middle part and yellow part of bamboo culm. For the whole lignin of syringyl and guaiacyl units on CC, it was the totally same with the distribution of lignin on SW from 12-day-old to 4-year-old.

In different development period of *Phyllostachys* *pubescens*, the lignification progress of SW and CC experienced from the green part of bamboo culm to the yellow part of bamboo culm from 17-day-old to 4-year-old samples, except it varied greatly on radial location when the fibre was 12 days old. In the middle and last period of fibre lignification, the lignification progress and degree were similar to each other on radial location. These results supported the conclusion which the lignification of fibre and parenchyma cell developed from the outside to inside of bamboo culm of *Phyllostachys pubescens* [13].

Along the radius bamboo culm the modulus, hardness and carbohydrates concentration also had a gradient trend [30]. The change trend of elastic modulus and hardness was positive correlation with lignin content, and the carbohydrate concentration was negative correlation with lignin content [12]. This is the relationship between the lignin distribution in micro-morphological regions and the macro properties of bamboo.

### 3.5. Variation of Lignin Content of Different Cell Wall Micro-Morphological Regions

**Figure 7:**
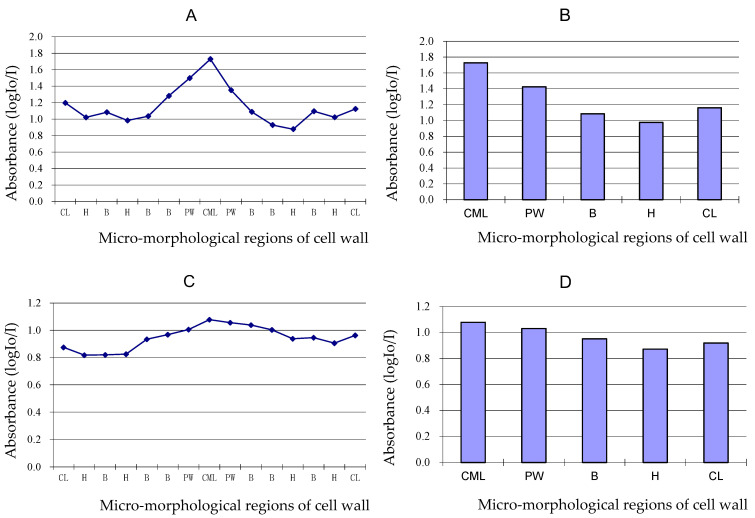
Curve and histogram of absorbance of Mäule (**A**,**B**) and Wiesner (**C**,**D**) reactions on different micro-morphological regions of four-year-old fibre wall in middle part of bamboo. CML, cell middle lamella, PW, primary wall; B, thin lamella of secondary wall; H, thick lamella of secondary wall; CL, cell lumen.

As shown in Figure 7A,B, the absorbance value showed syringyl lignin distribution on different micro-morphological regions of fibre cell wall in Mäule reaction when the fibre was four years old. The curve and histogram of absorbance showed the syringyl lignin content of CML was the most abundant, the syringyl lignin content of PW was the secondary, and the syringyl lignin content of SW was the least. Furthermore, the syringyl lignin content of thin layer of SW was always higher than that of thick layer of SW. Both single molecule lignin content of primary wall is higher than the secondary cell wall.

Figure 7C,D showed the both syringyl and guaiacyl lignin units distribution on different micro-morphological regions of fibre cell wall in Wiesner reaction when the fibre was four years old. As shown in the curve and histogram of absorbance, the trend of both syringyl and guaiacyl lignin distribution on different micro-morphological regions were similar to that of syringyl lignin distribution. The lignin content between two adjacent cells was the highest, followed by the cell lumen and, finally, the cell wall. It could be judged that the lignin monomer in the cell wall may come from the protoplast existing in the cell lumen, and the lignin monomer substances can permeate and flow between cells.

## 4. Conclusions

As one of the main components of the cell wall, lignin is distributed everywhere on different micro-morphological regions in the developmental fibre of *Phyllostachys pubescens*. Lignification develops by aging. Guaiacyl lignin units and syringyl lignin units can be found in the cell wall of the fibre, parenchyma, and vessel. The difference in lignin content among different ages, different radial location, and different micro-morphological regions of the cell wall was observed in this paper. The fibre walls were rich in guaiacyl lignin in the early stage of lignification, and lignin rich in syringyl units were deposited in the later stage. The guaiacyl and syringyl lignin deposition of bamboo green was earlier than that of the middle part of bamboo culm, and that of the middle part of bamboo culm was earlier than that of bamboo yellow. The multilayer of the fibre secondary cell wall has alternating thick and thin layers, while both single molecule lignin content of thin layer is higher than that of thick layers and the primary wall is higher than the secondary cell wall. This also shows that the rules of lignin deposition is consistent with the rules of cell wall formation. It is considered that lignin plays an important role in cell wall formation and cell wall mechanical properties. Lignin is related to the physical and mechanical properties of bamboo. Therefore, the study of the distribution and change of lignin in bamboo development is conducive to the mastery and prediction of various properties in the bamboo development, and has guiding significance for bamboo and lignin industrial utilization.

## Figures and Tables

**Table 1:** Absorption peaks with Mäule and Wiesner reactions of fibre.

Type of Reaction	Absorption Peak of Spectra (nm)			
	12-Day-Old	17-Day-Old	1-Year-Old	4-Year-Old
Mäule reaction	500	500	510	510
Wiesner reaction	515	505	500	520

## Data Availability

The data presented in this research are available on request from the corresponding author.
